# Not just a garbage truck: No-go decay plays a role during embryo development in zebrafish

**DOI:** 10.1371/journal.pbio.3002925

**Published:** 2024-12-06

**Authors:** Alana R. Plastrik, Hani S. Zaher

**Affiliations:** Department of Biology, Washington University in St. Louis, St. Louis, Missouri, United States of America

## Abstract

In eukaryotes, defective mRNAs that impede the movement of the ribosome are subject to rapid decay via no-go decay (NGD). In this issue of *PLOS Biology*, Ishibashi and colleagues expand on the role of NGD and reveal new endogenous targets for the process in zebrafish.

## Abstract 2

Vertebrate embryonic development is a complex process, involving several gene-regulatory strategies. This Primer explores a new study in PLOS Biology revealing the role of the mRNA-surveillance mechanism of no-go decay in regulating gene expression during embryonic development in zebrafish.

Efficient protein synthesis relies on the ability of ribosomes to smoothly move along the mRNA template during the elongation phase of translation. mRNA roadblocks impede ribosome transit and cause it to stall [1]. Persistent stalling eventually leads to upstream ribosomes colliding into the lead one [2]. These collisions can have detrimental impacts on cellular homeostasis. To cope with ribosome collisions, eukaryotes evolved ribosome quality control (RQC) to rescue ribosomes and target the nascent peptide for degradation [3]. The process is intimately coupled to the mRNA-surveillance mechanism of no-go decay (NGD), which targets the defective mRNA for degradation. Although the details by which downstream mRNA degradation occurs appear to be distinct between yeast and humans, the initiating signal for RQC and NGD is conserved [4]. Both processes are critically dependent on recognition of collided ribosomes by the E3 ligase ZNF598, which adds K63-linked ubiquitin chains to several ribosomal proteins [5]. Ubiquitinated ribosomal proteins recruit the ribosome splitting machinery (RQT/ASC1 complex), as well as mRNA decay factors (N4BP2/NONU-1/Cue2). Finally, the incomplete nascent peptide is presented to the proteasome for degradation following its ubiquitination by LISTERIN and release by ANKZF1 [6]. Widespread ribosome stalling exhausts the RQC machinery and activates the integrated stress response (ISR) and the ribotoxic stress response (RSR) [7]. ISR and RSR are activated by collided ribosomes through the kinases Gcn2 and ΖΑKα, respectively.

Although it has been well established that NGD targets defective mRNAs, including those damaged by alkylation and oxidizing agents [8], and UV irradiation [7]; studies on its role in regulating endogenous mRNAs have been limited. Notably, NGD is one of at least 3 mRNA-surveillance pathways that include nonsense-mediated decay (NMD) and non-stop decay (NSD). Interestingly, while initial studies on NMD focused on its role as a quality-control process targeting mRNAs harboring premature stop codons, we have come to appreciate that NMD is critical for cellular differentiation [9]. As a result, it is plausible that RQC/NGD have been repurposed to serve a role in gene expression. In support of this idea, studies in yeast and *Caenorhabditis elegans* have successfully identified endogenous targets for NGD. However, the extent to which the pathway regulates endogenous mRNA stability in vertebrates is poorly understood.

In this issue of *PLOS Biology*, Ishibashi and colleagues directly address this using zebrafish’s maternal to zygotic transition (MZT) [10], as this developmental stage is critically dependent on large-scale mRNA degradation. Previous studies had established that 3 mechanisms contribute to maternal mRNA decay during MZT, which include codon-optimality, micro-RNAs and m^6^A modification. Ishibashi and colleagues sought to expand on these mechanisms and asked whether NGD functions with these mechanisms to control mRNA stability during MZT. They took advantage of their previously generated *znf598* mutant strain, which is unable to ubiquitinate collided ribosomes and as a result defective in eliciting NGD. This mutant allowed the authors to ask which mRNAs are stabilized in the absence of ZNF598 activity during and right after fertilization. RNA-seq analysis revealed that about 200 mRNAs are stabilized relative to the wild type at 1 hour post fertilization (hpf); a similar number of mRNAs were stabilized at the 6 hpf mark. Notably, only ~50 of these mRNAs overlapped between the 2 developmental stages, suggesting that ZNF598 plays a role in regulating both maternal and zygotic mRNAs. More importantly, whereas no obvious functional enrichment could be detected for mRNAs that were stabilized at the 1 hpf in the *znf598* mutant strain, those stabilized at 6 hpf exhibited a significant enrichment for mRNAs containing the C2H2-type zinc finger domain (C2H2-ZF).

Highlighting the mechanistically distinct role of NGD in regulating mRNA stability during MZT, Ishibashi and colleagues found no significant overlap between its targets and those that have been annotated as targets for codon-optimality-, miRNA-, and m^6^A-based decay. Although, mRNAs encoding C2H2-ZF protein domains appear to be targeted for ZNF598-dependent degradation, whether decay is triggered through an NGD-based mechanism was unclear. Ishibashi and colleagues provided 3 lines of evidence to suggest that the mRNAs are indeed targets of NGD: (1) blocking of translation initiation stabilized them; (2) unlike the codon-optimality based mechanism, deadenylation appears to have no role in the decay process; (3) inhibition of eS10 ribosomal protein ubiquitination slows down decay.

Having established that C2H2-ZF-encoding mRNAs are likely NGD targets, Ishibashi and colleagues next sought out to verify that their decay is triggered through ribosome stalling. They utilized an mRNA-based reporter that encodes a *Renilla* luciferase (Rluc) and firefly luciferase (Fluc) separated by the ribosome skipping sequence P2A. Insertion of tandem C2H2-ZF-encoding regions from the *ZNF236* ORF between the 2 luciferases resulted in a significant decrease of Fluc luminescence relative to that of Rluc. Hence, C2H2-ZF domains slow translation elongation. Interestingly, and in contrast to what was observed in yeast and humans, inactivation of ZNF598 did not lead to readthrough on these tentative stall sequences, but instead it reinforced stalling on them. In particular, injection of *znf236* mRNA into *MZznf598* embryos produced more abortive protein products and significantly less full-length one relative to injections into wild-type embryos. Finally, using ribosome profiling and a new disome score, Ishibashi and colleagues revealed that decay of C2H2-ZF-encoding mRNAs correlates with disome peaks. In conclusion, C2H2-ZF-encoding mRNAs appear to be prone to stalling and eventual ribosome collisions which in turn initiate their degradation through NGD.

This new study sheds some important insights into the potential role of a quality control process in vertebrate development. Nevertheless, several questions regarding the specificity, mechanism, and utility of NGD in regulating gene expression remain unanswered (**Fig 1**). Chief among these questions is how conserved this mode of regulation is. The authors attempted to address this in human cells; and while they observe increased stalling on C2H2-ZFs when inserted in a dual luciferase reporter, ZNF598 deletion did not alter the decay rate of the endogenous mRNAs. It is possible that NGD targets a different class of mRNAs in humans depending on the cell type and developmental stage; more careful studies are needed to understand the role of NGD in other vertebrates. Equally important is whether and how RQC and NGD are regulated throughout development and differentiation. More specifically, is the activity of ZNF598 regulated through its expression and/or posttranslational mechanisms? Similarly, what features of C2H2-ZF-encoding mRNAs are responsible for stalling? Ishibashi and colleagues suggested that the positive charge of the nascent peptide and the codon context around the C2H2-ZF regions can partially explain their stalling behavior. However, it is clear that stalling on these sequences is more complex than that. It would be interesting to assess the role of co-translational folding and the associated chaperones in stalling. Perhaps, the most important question yet to be addressed is how important this mode of regulation is in cellular differentiation. Zebrafish MZT appears to occur just fine in the absence of ZNF598 and downstream NGD, suggesting that the process is not critical for MZT. And while young adult animals are smaller relative to wild-type ones, this phenotype appears to be the result of overactivation of the ISR, and not overabundance of NGD targets per se. More work is clearly needed to completely understand the functional relevance of RQC and NGD in other species, and how their activities intersect with other pathways such as the ISR and RSR [7].

**Fig 1:**
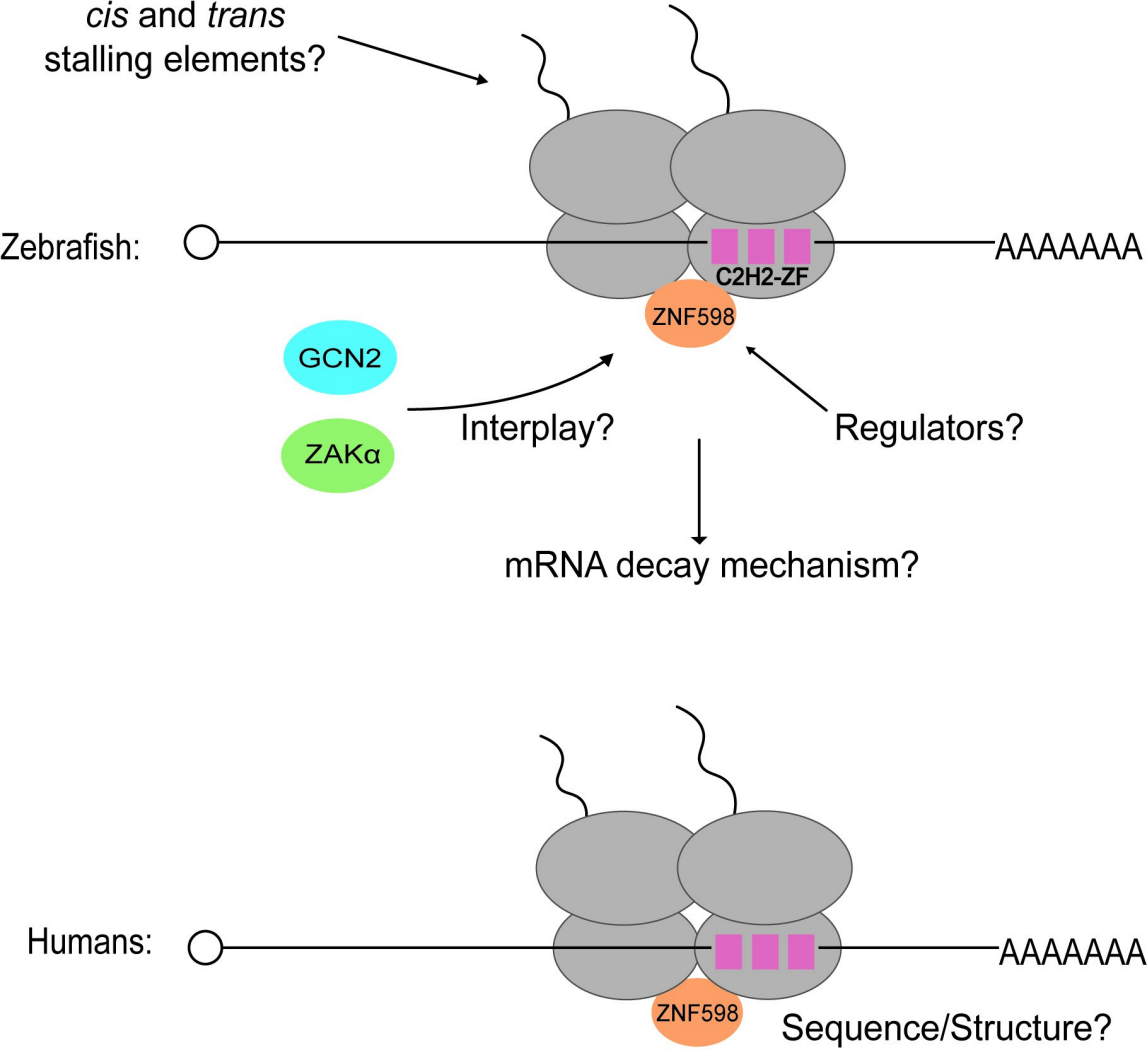
C2H2-ZF-encoding mRNAs stall ribosomes and are subject to ZNF598-mediated NGD in zebrafish. A complete understanding of the factors that slow down translation on these motifs is missing. So is how the process might be regulated. Similarly, how other collision-sensing mechanisms (GCN2 and ZAKα) impact ZNF598’s function remains unclear. Whether ZNF598 is regulated during development requires further investigation. The mechanism by which the mRNAs are degraded has not been rigorously explored. Finally, the endogenous targets for NGD in humans are yet to be identified.
